# Clinical significance of hypoalbuminemia in outcome of patients with scrub typhus

**DOI:** 10.1186/1471-2334-10-216

**Published:** 2010-07-21

**Authors:** Chang-Seop Lee, In-Suk Min, Jeong-Hwan Hwang, Keun-Sang Kwon, Heung-Bum Lee

**Affiliations:** 1 Department of Internal Medicine, Chonbuk National University Medical School and Research Institute of Clinical Medicine, Geumam-dong, Jeonju, 561180, Republic of Korea; 2 Chonbuk National University Medical School, Geumam-dong, Jeonju, 561180, Republic of Korea; 3 Department of Internal Medicine, Chonbuk National University Medical School, Geumam-dong, Jeonju, 561180, Republic of Korea; 4 Preventive Medicine, Chonbuk National University Medical School and Research Institute of Clinical Medicine, Geumam-dong, Jeonju, 561180, Republic of Korea

## Abstract

**Background:**

This study was designed to investigate the clinical significance of hypoalbuminemia as a marker of severity and mortality in patients with Scrub typhus.

**Methods:**

The patients with scrub typhus were divided into two groups based on the serum albumin levels; Group I (serum albumin <3.0 g/dL) and Group II (serum albumin ≥3.0 g/dL). The outcome of patients with hypoalbuminemia was compared with that of normoalbuminemia.

**Results:**

Of the total 246 patients who underwent the study, 84 patients (34.1%) were categorized as Group I and 162 patients were (65.9%) as Group II. Group I showed significantly higher incidence of confusion (24.6% vs. 5.3%, *p *< 0.001), pulmonary edema (15.8% vs. 3.2%, *p *= 0.002), pleural effusion (22.8% vs. 11.1%, *p *= 0.03), arrhythmia (12.3% vs. 2.6%, *p *= 0.008) and non-oliguric acute renal failure (40.4% vs. 11.1%, *p *< 0.001) compared to group II. Hypoalbuminemic group had a higher APACHE II score (11.37 ± 5.0 vs. 6.94 ± 4.2, *p *< 0.001), longer hospital stay (19.9 ± 42.1 days vs 7.5 ± 13.8 days, *p *= 0.012), and higher hospital cost compared to Group II.

**Conclusions:**

This study showed hypoalbuminemia in scrub typhus was closely related to the frequency of various complication, longer hospital stay, consequently the higher medical cost, necessitating more efficient management of patients, including medical resources.

## Background

Scrub typhus is an acute febrile illness with the characteristic findings of high fever, eschar, maculopapular rash, lymphadenopathy, headache, and myalgia [1-3]. Scrub typhus caused by infection with *Orientia tsutsugamushi *occurs over a wide area of Eastern Asia and the Western Pacific region [1]. Usually, the symptoms of this disease are mild and its clinical course is uneventful. However, some patients experience severe or fatal events such as acute renal failure, respiratory distress or multiorgan dysfunctions [1,4,5].

Generally, hypoalbuminemia is known to be associated with complications and mortality in patients with acute infectious disease [6]. In scrub typhus, about 25%~69.2% of patients presented hypoalbuminemia [7,8]. For the mechanism of hypoalbuminemia, decreased synthesis of albumin due to hepatic dysfunction, increased catabolism of protein, albuminuria, decreased intestinal absorption of protein due to poor oral intake, and extensive vascular leakage of serum protein due to increased capillary permeability have been postulated [7].

Despite the common occurrence of hypoalbuminemia in patients with scrub typhus, there has been limited information about the relationship between the hypoalbuminemia and the severity of disease. To confirm the significance of hypoalbuminemia on the outcome of patients with scrub typhus, the clinical records of 299 patients with Scrub typhus were reviewed retrospectively.

## Methods

### Study population and design

**Figure 1:**
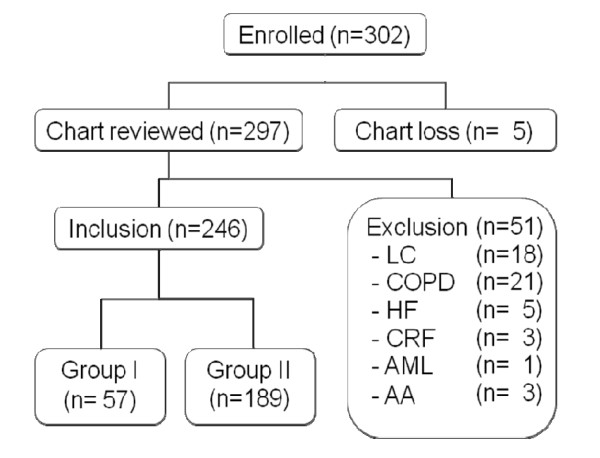
**Flow diagram for study subjects**. LC, liver cirrhosis; COPD, chronic obstructive pulmonary disease; HF, heart failure; CRF, chronic renal failure; AML, acute myelogenous leukemia; AA, aplastic anemia.

A total of 302 cases (age ≥18 years) who were admitted from January, 2001 through December, 2006 at Chonbuk National University Hospital, Jeonju, South Korea, and had a history of fever with either eschar or a maculopapular skin eruption and more than 2 vague symptoms (such as headache, malaise, myalgia, coughing, nausea, and abdominal discomfort) were serologically titrated and diagnosed as scrub typhus. However, 56 cases were excluded from the study because of lost records (n = 5), or the presence of underlying diseases (liver cirrhosis, n = 18; chronic obstructive pulmonary disease, n = 21; heart failure, n = 5; chronic renal failure, n = 3; acute myelogenous leukemia, n = 1; aplastic anemia, n = 3). The remaining 246 patients were enrolled (Figure 1). In this study, we excluded the patients with underlying diseases because the albuminemia in these cases was considered due to the host factor, which might be associated with the poor prognosis. The outcome of patients with hypoalbuminemia was compared with that of normoalbuminemia. For the purpose of this study, patients with scrub typhus with serum albumin of <3.0 g/dL were defined as 'Group I', while those with serum albumin of ≥3.0 g/dL were defined as 'Group II'. The albumin level was checked on the first blood samples taken upon their visit to the hospital.

### Diagnosis of scrub typhus

The diagnosis of scrub typhus was made on clinical manifestations and passive hemagglutination assay (PHA) against *O. tsutsugamushi*. A definite case of scrub typhus was defined by an increased titer of Passive Hemagglutination Assay (PHA) against *O. tsutsugamushi *(≥1:80) in a single serum sample or by a 4-fold or greater increase of titer in the follow-up [9-11]. PHA was performed at the NeoDIN Medical Institute in Seoul, Korea using Genedia Tsutsu PHA II test kits. Genedia Tsutsu PHA II is a test kit for the qualitative and quantitative detection of antibodies against *O. tsutsugamushi *in human serum based on the PHA. In the kit, we used sheep erythrocytes sensitized by *Karp *and *Gilliam *strains, including the *Boryong *strain found in Korea [12,13].

### Definitions

Hypoalbuminemia was defined as a first serum albumin of less than 3.0 g/dL on their initial visit [6,8]. Central nervous system (CNS) involvement was defined by the presence of CNS related symptoms such as confusion, seizure or coma. Involvement of respiratory system (RS) was defined by the presence of symptoms, signs and investigation results supporting evidence of pulmonary edema, interstitial pneumonia, pleural effusion or the need for mechanical ventilation. Cardiovascular system (CS) involvement was defined by the presence of symptoms, signs and investigation results supporting pericardial effusion, new onset of atrial fibrillation and ischemic heart disease. New onset of atrial fibrillation was defined as such when the symptoms improved with clinical recovery of scrub typhus. Gastrointestinal system (GS) involvement was defined by the presence of symptoms, signs and investigation results supporting gastric ulcer, pancreatitis, and upper gastrointestinal bleeding. Acute renal failure (ARF) was defined as such when at least 50% reduction was seen in glomerular filtration rate (GFR) using the abbreviated modified diet in renal disease (MDRD) equation, namely: GFR(mL/min/1.73 m^2^) = 186 P_cr_^-1.154 ^× age^-0.203 ^× (1.212 if black) × (0.742 if female) [14]. An "ST interval" was defined as an interval from the day when clinical symptoms began (prior to the admission) and to the day when appropriate treatment with antibiotics (Doxycycline or azithromycin) begun. An "AT interval" was defined as an interval from the day of admission to the day when appropriate treatment started.

### Costs

All costs were expressed in US dollars. The currency rate was 1,300 Korean Won per 1 United States Dollar. We estimated only the direct medical cost, which included treatment (oral and/or intravenous administration), examination (laboratory and imaging studies), surgical and nonsurgical procedures, physical therapies, and room and board.

### Statistical analysis

The results were analyzed using SPSS v 15.0 (SPSS, Inc., Chicago, IL, USA). Categorical variables were compared by Fisher's exact test or chi-square test and continuous variables were compared by the ANCOVA. All tests of significance were two-tailed; p values ≤ 0.05 were considered to be significant.

## Results

Since the age in Group I was significantly older than Group II (*p *< 0.001), we adjusted all variables for age to avoid biased outcome due to this demographic difference. Meanwhile, there were no statistical differences in gender and underlying diseases such as diabetes mellitus, hypertension, chronic hepatitis B and chronic hepatitis C.

As it might be expected from the symptoms and signs, hypoalbuminemic patients had much higher incidence of dyspnea (*p *< 0.001) compared to Group II, but the presence of typical signs of scrub typhus such as skin rash, eschar formation and lymphadenopathy and other vague complaints were not significant (Table 1). According to the rate of organ dysfunction, the rate of CNS (*p *= 0.006), RS (*p *< 0.013), GS (*p *= 0.012), and non-oliguric ARF (*p = *0.001) were higher in Group I than in those of Group II, while the incidence of CS (*p *= 0.299) differed not significantly between the two groups (Table 2).

**Table 1:** Demographic and Clinical Characteristics of 246 Study Patients

Characteristics	Group I	Group II	***P ***^**†**^
Age, mean ± SD, years	71.4 ± 10.6	60.7 ± 12.2	<0.001
Sex			
Male	22 (38.6)	62 (32.8)	0.311
Female	35 (61.4)	127 (67.2)	
Underlying disease			
Diabetes Mellitus	2 (3.5)	1 (0.5)	0.660
Hypertension	16 (28.1)	45 (23.8)	0.734
Chronic hepatitis B	0 (0.0)	5 (2.6)	0.997
Chronic hepatitis C	2 (3.5)	1 (0.5)	0.288
Clinical manifestations			
Chills	47 (82.5)	166 (87.8)	0.658
Sore throat	9 (15.8)	30 (15.9)	0.453
Headache	35 (61.4)	124 (65.6)	0.566
Myalgia	38 (66.7)	117 (61.9)	0.546
Arthralgia	0 (0.0)	11 (5.8)	0.997
Cough	20 (35.1)	66 (34.9)	0.600
Dyspnea	26 (45.6)	38 (20.1)	0.012
Nausea/vomiting	24 (42.1)	71 (37.6)	0.297
Abdominal pain	24 (42.1)	55 (29.1)	0.103
Skin rash	38 (66.7)	123 (65.1)	0.639
Eschar	42 (73.7)	131 (69.3)	0.191
Lymphadenopathy	3 (5.3)	20 (10.6)	0.434
Conjunctivitis	5 (8.8)	24 (12.7)	0.759

^† ^Adjusted for age; SD, standard deviation.

**Table 2:** Complications of the Patients in the study

Complications	Group I	Group II	***P ***^**†**^
Central Nervous System	17 (29.8)	14 (7.4)	0.006
Confusion	14 (24.6)	10 (5.3)	0.021
Seizure	2 (3.5)	1 (0.5)	0.191
Coma	1 (3.5)	3 (1.6)	0.814
Respiratory System	26 (45.6)	38 (20.1)	0.013
Pulmonary edema	9 (15.8)	6 (3.2)	0.002
Pleural effusion	13 (22.8)	21 (11.1)	0.162
Pneumonia	1 (1.8)	9 (4.8)	0.044
Ventilator use	3 (5.3)	2 (1.1)	0.141
Cardiovascular System	9 (15.8)	10 (5.3)	0.299
Pericardial effusion	1 (1.8)	1 (0.5)	0.456
Arrhythmia*	7 (12.3)	5 (2.6)	0.215
Ischemic heart disease	1 (1.8)	4 (2.1)	0.561
Gastrointestinal System	10 (17.5)	10 (5.3)	0.012
Peptic ulcer	6 (10.5)	7 (3.7)	0.226
Pancreatitis	1 (1.8)	0 (0.0)	0.995
Upper gastrointestinal bleeding	2 (3.5)	0 (0.0)	0.995
Cholecystitis	0 (0.0)	2 (1.1)	0.997
Appendicitis	1 (1.8)	1 (0.5)	0.168
Kidney	27 (47.4)	27 (14.3)	<0.001
Non-oliguric ARF	23 (40.4)	21 (11.1)	0.001
Oliguric ARF	4 (7.0)	6 (3.2)	0.561

^† ^Adjusted for age; ARF, acute renal failure.

*Arrhythmia; new onset of atrial fibrillation.

A summary of initial laboratory findings for groups I and II is shown in Table 3. The serum albumin levels on admission in Group I and Group II were 2.69 ± 0.22 and 3.56 ± 0.35 g/dL, respectively. In Group I, leukocyte count (*p *= 0.007), AST (*p = *0.024) and total bilirubin (*p *< 0.001), and BUN were higher, and platelet count was significantly lower (*p *< 0.001) compared to Group II. On the other hand, there were no statistical differences in hematocrit, hemoglobin, ALT, BUN, Na^+ ^, and K^+ ^between the two groups. The GFR (*p *= 0.050) was marginally lower in Group I than in Group II. The ST (*p = *0.406) and AT (*p = *0.487) interval was not significantly different between the two groups. Although the patients of Group I had higher APACHE II score (*p *< 0.001), medical costs (*p *< 0.001) and longer hospital stay (*p *< 0.001) than the Group II, there was no difference in mortality (*p *= 0.796) (Table 4, 5).

**Table 3:** Admission Laboratory Findings of the study Patients*

	Group I	Group II	***P ***^**†**^
WBC (10^3^/μL)	10.79 ± 4.04	8.57 ± 4.10	0.007
Hematocrit (%)	30.87 ± 10.98	31.86 ± 12.03	0.869
Hemoglobin (g/dL)	11.63 ± 1.92	12.38 ± 1.60	0.067
Platelet (10^3^/mm^3^)	115.11 ± 69.07	161.28 ± 90.92	< 0.001
AST (IU/L)	121.60 ± 85.95	106.87 ± 99.44	0.024
ALT (IU/L)	107.35 ± 168.92	97.15 ± 103.94	0.084
T-bili (mg/dL)	1.417 ± 1.33	0.84 ± 0.59	<0.001
Albumin (g/dL)	2.69 ± 0.22	3.56 ± 0.35	<0.001
BUN (mg/dL)	32.94 ± 17.05	19.50 ± 14.11	<0.001
GFR (mg/dL)	61.64 ± 37.37	79.51 ± 33.16	0.050
Na^+ ^(mmol/L)	134.18 ± 6.11	135.19 ± 11.14	0.573
K^+ ^(mmol/L)	3.70 ± 0.70	3.88 ± 0.71	0.109

* Values are the mean ± standard deviation; ^† ^Adjusted for age; WBC, white blood cell; AST, aspartate aminotransferase; ALT, alanine aminotransferase; BUN, blood urea nitrogen; GFR, glomerular filtration rate; Na^+^, sodium; K^+^, potassium.

**Table 4:** The Comparison of ST, AT interval, APACHE II score, Intensive Care Unit Admission, Septic Shock, and Mortality of the Patients*

	Group I	Group II	***P ***^**†**^
ST interval, mean ± SD	6.84 ± 4.14	7.44 ± 4.12	0.406
AT interval, mean ± SD	0.98 ± 1.89	0.87 ± 2.34	0.487
APACHE II Score, mean ± SD	11.37 ± 5.1	6.94 ± 4.2	<0.001
Intensive Care Unit Admission	10 (17.5%)	13 (6.9%)	0.162
Septic Shock	11 (19.3%)	15 (7.9%)	0.134
Mortality	3 (5.3%)	6 (3.2%)	0.796

* ST interval = from the day when clinical symptoms began and to the day when appropriate treatment with antibiotics began; AT interval = from the day of admission to the day when appropriate treatment started.

^† ^Adjusted for age; APACHE II, Acute physiology and chronic health evaluation; SD, standard deviation

**Table 5:** Comparison of Length of hospital Stay, and the Cost Between the Two Groups*

	Group I	Group II	*P*
Duration of hospital Stay, mean ± SD	11.46 ± 8.9	5.93 ± 5.9	<0.001^†^
Cost (USD)	2357.83 ± 2284.15	1135.97 ± 1668.62	<0.001

^† ^Adjusted for age; SD, standard deviation; 1 USD (united states dollar); 1,300 Won (Korean Currency).

## Discussion and Conclusion

In this study, scrub typhus patients with hypoalbuminemia had a higher APACHE II score, longer hospital stay, and higher medical cost compared to the patients without hypoalbuminemia. And in the hypoalbuminemic group, thrombocytopenia and leukocytosis, which were known to be clinical parameters representing disease severity [15], were more severe. In addition, the hypoalbuminemic group showed a higher rate of complication in CNS, RS, GS, and non-oliguric ARF.

Generally, the mechanism of hypoalbuminemia in acute infectious disease is known to be related with poor oral intake of protein, decreased synthesis of protein from the liver, increased catabolism of protein, and increased metabolism of albumin due to the vascular leakage of serum protein due to increased vascular permeability [16]. A major and serious pathologic change of scrub typhus is a focal or disseminated vasculitis due to the destruction of endothelial cell lining of the small vessels, which is manifested as perivascular infiltration of leukocytes [17-19], an increased vascular permeability with extravascular protein loss, and consequently the hypoalbuminemia.

The widespread vasculitis or perivasuculitis in scrub typhus may involve the lung, cardiovascular system, brain, kidney, gastrointestinal tract, liver, and lymph nodes [4,17-21]. Song *et al *[8] showed that interstitial pneumonia, pleural effusion, and pulmonary edema were more frequently reported in scrub typhus patients with hypoalbuminemia than in patients without hypoalbuminemia. In this study, dyspnea and pulmonary edema occurred more frequently in the group with hypoalbuminemia than in the patients without hypoalbuminemia. This finding concurred with that of Song et al [8].

Previous reports showed that septic shock [22] and hepatic dysfunction [23] were frequently reported in patients with scrub typhus with hypoalbuminemia. They suggested that these complications were closely associated with the disease severity of scrub typhus. In this study, the level of AST and total bilirubin were higher in the hypoalbuminemia group than in the group without hypoalbuminemia.

Acute renal failure can occur due to acute tubular necrosis caused by direct invasion of *Oriential tsutsugamushi *[24]. Renal complications may prolong its morbidity and even lead to death. In this study, renal failure was more frequently reported in the group with hypoalbuminema. We suggest that the hypoalbuminemia has close association with acute renal failure. The absolute WBC counts varied but had a significantly higher mean value in the hypoalbuminemia group. This suggests that patients with hypoalbuminemia might have been more seriously ill.

Though hypoalbuminemia is a powerful predictor of mortality in patients with various illnesses [25-27], this study did not show significantly higher mortality in patients with hypoalbuminemia than the nonhypoalbuminemic patients. Incidence of severe scrub typhus is actually low, and an active treatment with antibiotics will suffice for managing the case. Therefore, the hypoalbuminemia itself may be insignificant in predicting poor clinical courses in scrub typhus. This suggestion can be supported by lower APACHE II score in hypoalbuminemic scrub typhus patients compared to other severe septic conditions.

The length of hospital stay in the hypoalbuminemic group was significantly longer than that of group without hypoalbuminemia, raising the cost of their hospital stay significantly higher for more tests and treatments. It is suggested that scrub typhus patients with hypoalbuminemia had more complications than the patients without hypoalbuminemia, although their mortality rate remained low.

Our study has some limitations. Firstly, our study is retrospective, which has obvious limitations. Secondly, we classified the level of albumin into two groups. Therefore, the clinical impact of hypoalbuminemia on patients with scrub typhus could not be evaluated. Thirdly, outcomes of albumin administration had not been evaluated.

As the annual incidence of scrub typhus in Korea has been increasing steadily with its reported cases sharply rose to 6057 in 2008 [28], the financial burden of caring for scrub typhus is becoming an emerging problem. This study was done in search of more effective measures for improving patient care in scrub typhus. The hypoalbuminemia as a criterion is an important marker on the clinical outcome of patients with scrub typhus.

## Competing interests

The authors declare that they have no competing interests.

## Authors' contributions

CSL contributed to the concept, design, conduct and analysis of the study and also wrote the manuscript. ISM contributed to the study design, conduct and also wrote the manuscript. JHH contributed to the study concept, design and interpretation and provided a critical review of the manuscript. KSK contributed to the design, implementation of the study, provided an oversight of data collection and statistical analysis. HBL contributed to the design and interpretation of the results and a critical review of the manuscript. All authors read and approved the final manuscript.

## Pre-publication history

The pre-publication history for this paper can be accessed here:

http://www.biomedcentral.com/1471-2334/10/216/prepub
